# Cellular Stress and p53-Associated Apoptosis by *Juniperus communis* L. Berry Extract Treatment in the Human SH-SY5Y Neuroblastoma Cells

**DOI:** 10.3390/ijms17071113

**Published:** 2016-07-13

**Authors:** Tiina A. Lantto, Into Laakso, H. J. Damien Dorman, Timo Mauriala, Raimo Hiltunen, Sulev Kõks, Atso Raasmaja

**Affiliations:** 1 Division of Pharmaceutical Biosciences, Faculty of Pharmacy, University of Helsinki, Post Office Box 56 (Viikinkaari 5E), Helsinki 00014, Finland; tiina.lantto@helsinki.fi (T.A.L.); into.laakso@helsinki.fi (I.L.); damien.dorman@helsinki.fi (H.J.D.D.); timo.mauriala@orion.fi (T.M.); raimo.hiltunen@helsinki.fi (R.H.); 2 Department of Pathophysiology, Institute of Biomedicine and Translational Medicine, University of Tartu, 19 Ravila Street, Tartu 50411, Estonia; sulev.koks@ue.ee; 3 Division of Pharmacology & Pharmacotherapy, Faculty of Pharmacy, University of Helsinki, Post Office Box 56 (Viikinkaari 5E), Helsinki 00014, Finland; 4 Department of Physiology, Institute of Biomedicine and Translational Medicine, University of Tartu, 19 Ravila Street, Tartu 50411, Estonia

**Keywords:** apoptosis, *Juniperus communis* L., plant extract, ER stress, p53, SH-SY5Y neuroblastoma cells

## Abstract

Plant phenolics have shown to activate apoptotic cell death in different tumourigenic cell lines. In this study, we evaluated the effects of juniper berry extract (*Juniperus communis* L.) on p53 protein, gene expression and DNA fragmentation in human neuroblastoma SH-SY5Y cells. In addition, we analyzed the phenolic composition of the extract. We found that juniper berry extract activated cellular relocalization of p53 and DNA fragmentation-dependent cell death. Differentially expressed genes between treated and non-treated cells were evaluated with the cDNA-RDA (representational difference analysis) method at the early time point of apoptotic process when p53 started to be activated and no caspase activity was detected. Twenty one overexpressed genes related to cellular stress, protein synthesis, cell survival and death were detected. Interestingly, they included endoplasmic reticulum (ER) stress inducer and sensor *HSPA5* and other ER stress-related genes *CALM2* and *YKT6* indicating that ER stress response was involved in juniper berry extract mediated cell death. In composition analysis, we identified and quantified low concentrations of fifteen phenolic compounds. The main groups of them were flavones, flavonols, phenolic acids, flavanol and biflavonoid including glycosides of quercetin, apigenin, isoscutellarein and hypolaetin. It is suggested that juniper berry extract induced the p53-associated apoptosis through the potentiation and synergism by several phenolic compounds.

## 1. Introduction

Cell signaling pathways related to apoptosis and cell cycle have an essential role in development and progression of complex diseases such as cancer. One of the key orchestrators of those cellular functions is the tumour protein p53 [1]. The fact that p53 dysfunctions in most cancers indicates its essential role in tumour suppression: p53 has been found to be mutated in half of the cases while other cases often possess dysregulation of its upstream signaling pathways [2]. Cellular stress, including DNA damage, ribosomal and endoplasmic reticulum stress activates p53 whereas its levels are strictly maintained low under normal conditions. Activated p53 translocates into the nucleus where it modulates the expression of over hundred genes [3]. Furthermore, p53 acts in cytoplasm where it regulates the mitochondrial membrane permeabilization and directly interacts with other proteins [4,5]. In addition to these well-known tumour suppressing activities of p53, recent studies have revealed that it has a central role in tumour-related metabolism, cell-cell communication and metastasis as well [1].

Juniper (*Juniperus communis* L., Cupressaceae) is an evergreen coniferous shrub or small tree growing on the temperate regions of the northern hemisphere. Mature female cones of juniper are generally called berries for their “berry-like” appearance, and they are used to flavour game meat and alcoholic beverages, e.g., gin and beer. In traditional herbal medicine juniper has been used for many purposes as e.g., treating wounds, pain, fevers, rheumatism, snakebites, swellings, gastrointestinal infections, bronchitis and cancers [6,7] and it has been claimed to possess also diuretic, antiseptic, carminative, stomachic and antirheumatic properties [8].

Although juniper berries are mainly used for their aromatic properties, they also contain bioactive plant phenolics, e.g., quercetin glycosides [9], which might explain at least some of the claimed health-promoting effects of juniper. It is known that naturally derived phenolic compounds can affect different cell signalling pathways inducing both cell cycle progression and apoptosis [10]. The mechanisms of single compounds have been studied more closely but the interest on the mixtures of compounds or plant extracts has been raised over the last years [11]. Suggested benefits of using combinations of different therapeutic agents include reduced toxicity based on the lower-dose usage of drugs and decreased development of drug resistance [12].

We have shown earlier that the juniper berry extract can induce a p53-dependent cell death in human SH-SY5Y neuroblastoma cells [13]. In addition, the anti- and pro-oxidant capacities of the extract have been analysed in biochemical test models [14]. In these studies, the juniper berry extract was prepared using a hydrodistillation process to remove volatile compounds [15], and their absence was verified with chromatographic analysis. Therefore, the observed anti- and/or pro-oxidant and cell death-inducing effects of the extract did not result from toxicity of volatile compounds but rather by specific non-volatile compounds mediated cellular mechanisms [13,14]. Studies on the bioactivity of juniper berry extracts without volatile components are rare in literature.

In the present work, we have studied the phenolic composition and biological effects of aqueous juniper berry extract in more detail. Therefore, we have examined the mechanisms of juniper extract induced apoptosis by analyzing the p53 translocation, and gene expression and DNA fragmentation. The identification and quantification of phenolic compounds was performed using chromatographic methods. The results showed that the juniper extract contained several compounds with cell cycle and apoptosis regulating effects. The extract treatment of human SH-SY5Y neuroblastoma cells resulted in the increased DNA damage, p53 translocation from the cytoplasmic to nuclear compartment and overexpression of several genes in parallel with the reactivation of impaired apoptosis.

## 2. Results

### 2.1. Phenolic Composition of Aqueous Juniper Berry Extract

**Figure 1:**
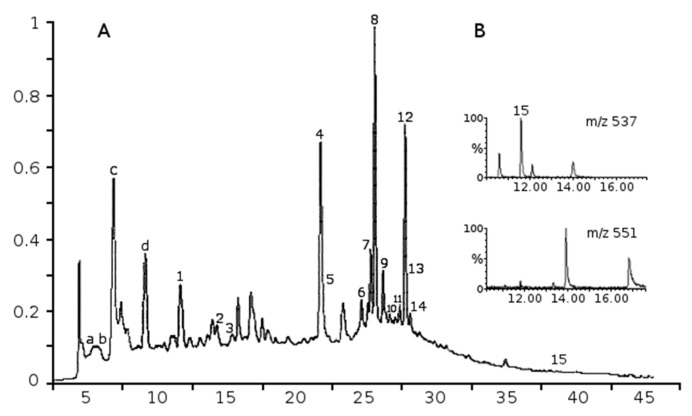
Analysis of the compounds of Juniper communis extract. (**A**) LC-MS-UV-chromatogram of water extract of juniper berries (280 nm); compound (a) *m*/*z* 191 (100), 353 [M − 1]; (b) *m*/*z* 169 [M − 1]; (c) *m*/*z* 539 (100); (d) *m*/*z* 443 (100); and (**B**) the iongram profiles of biflavones at *m*/*z* 537 and 551 obtained by quadrupole time of flight-electrospray ion (QTOF-ESI)-analysis. Numbered peaks see Table 1. *X*-axis is arbitary units, *Y*-axis is time in min.

Thirteen flavonoid glycosides and two phenolic acids (procatechuic acid and rosmarinic acid) were identified and quantified from the extract (Figure 1A, Table 1). Flavonoids included five flavonols (gossypetin-hexoside-pentoside, hyperoside, kaempferol-3-*O*-glucoside, quercetin pentoside, rutin); six flavones (apigenin-7-*O*-glucoside, isoscutellarein-8-*O*-pentoside, isoscutellarein-7-*O*-pentoside, hypolaetin hexoside, hypolaetin-7-*O*-pentoside, luteolin) and one flavanol (catechin). Among biflavonoids, only a small amount of amentoflavone was detected. However, traces of five other biflavonoids were found in quadrupole time of flight (QTOF) analyses by single ion monitoring at *m*/*z* 537 [M − 1] and at *m*/*z* 551 [M − 1] (Figure 1B). In addition, numerous polar compounds eluting at shorter retention times were characteristic in the LC-MS-UV analysis, but those compounds remained unidentified (a–d, Figure 1A). Overlapping peaks such as 4 and 5 (Figure 1) were separated into two compounds rutin *m*/*z* 609 [M − 1] and hyperoside *m*/*z* 463 [M − 1] by ultra-performance liquid chromatograhy (UPLC)-QTOF analysis. Free aglycones such as apigenin and quercetin or volatile oils components were not detected in extract.

### 2.2. Identified Phenolics and Concentrations

The content of total phenols determined as gallic acid equivalents accounted for 18.5 mg/g dried juniper berry extract [15]. Identified and quantified phenolic compounds comprised 42% of reported total phenols content, and they represented 7733 µg/g (0.77%) of the dry weight of extract. Flavones (flavonoids) comprised 54% of identified compounds, flavonols (flavonoids) 32%, phenolic acids 7%, flavanol (flavonoid) 5% and biflavonoid 2%. The main identified compounds were quercetin glycosides (27% of identified compounds), apigenin glycoside (21%), isoscutellarein glycosides (16%) and hypolaetin glycosides (15%). The four most abundant flavonoid glycosides (800–1650 µg/g) were apigenin-7-glucoside, rutin, hypolaetin-7-pentoside and isoscutellarein-7-*O*-pentoside. Concentrations of single compounds in cell treatments with 10 µg/mL of extract were calculated according to molecular weight data obtained from phenolic composition analysis (Table 1). Concentrations of single compounds in treatments varied from 2.53 to 38.10 nM.

### 2.3. Localization of Protein p53 in Cytoplasm and Nucleus

**Figure 2:**
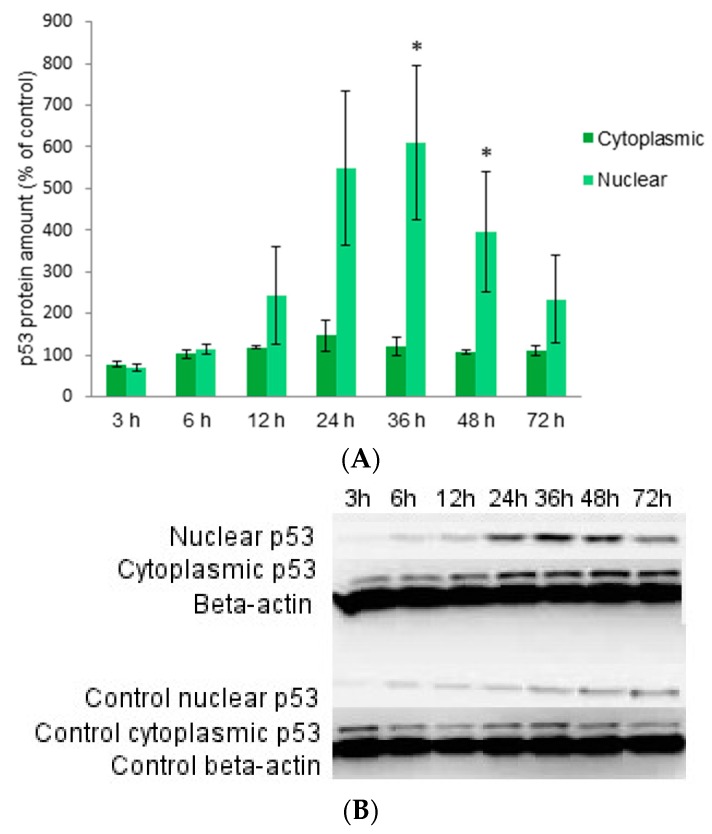
The amount of protein p53 in cytoplasmic and nuclear fractions of SH-SY5Y neuroblastoma cells at different time points after treatment with 10 μg/mL of juniper berry extract (**A**); The western blot analysis of p53 amount at different time points (**B**). Values are means ± SEM of protein amounts of treated cells compared to the untreated control cells from three independent experiments. Statistical significance was determined using a one-tailed Student’s *t*-test; * *p* < 0.05.

The accumulation of p53 after a 12 h-treatment with the juniper berry extract in human neuroblastoma SH-SY5Y cells was about two fold, i.e., in the same level than in our previous study [13] with statistically significant increases. Here we show that the accumulated p53 starts to translocate into the nucleus 12 h after treatment, and the highest nuclear p53 concentrations were detected after 24, 36 and 48 h (Figure 2). Translocation of p53 declines after 72 h of treatment. Observations were statistically significant after 36 and 48 h of treatment. The amount of cytoplasmic p53 is stable within the treatments when compared to the untreated control cells although the variation of nuclear amounts was detected between treatments. These observations suggest the transcriptional activation of p53 after treatment with juniper berry extract.

### 2.4. DNA Fragmentation and Morphology

**Figure 3:**
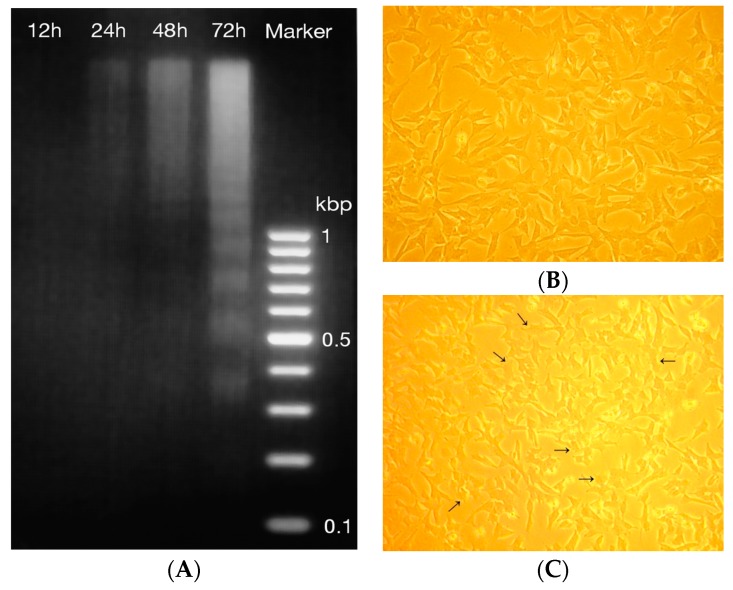
Features of cell death in neuroblastoma SH-SY5Y cells after treatment with 10 μg/mL of juniper berry extract. Fragmented DNA at different time points after treatment (**A**); Non-treated (**B**) and treated cells (**C**) after 12 h-treatment. The amount of 56% (SD 2.5, median 56, *n* = 3) of treated cells were morphologically slightly changed after treatment. The arrows point the slight shrinkage and membrane disruption of treated cells compared to non-treated cells. Original magnification of cells was 10×.

Fragmented DNA was detected first after 24 h-treatment but the most abundant laddering was detected after a 72 h-treatment (Figure 3A). The DNA of non-treated control cell was also analyzed (results not shown) and some minor breakage of DNA was detected later after 48 and 72 h but not 24 h after treatment. In addition, the visible DNA ladders were remarkably weaker compared to ladders detected from treated cells. Morphological changes in the treated and non-treated cells were observed by a microscopic examination. After the 12 h-treatment before the cell death occurred, the amount of 56% (SD 2.5, median 56) of treated cells were slightly shrunk and membranes slightly disrupted compared to the non-treated control cells but the amount of floating dead cells was comparable to the non-treated cells (Figure 2B,C). The detachment of cells increased gradually 24, 36, 48 and 72 h after the treatment. The breakage of DNA is one of the final steps of apoptotic cell death [26], and it is described as a hallmark of apoptosis [27]. These results suggest that the juniper berry extract induced the apoptotic cell death after 24 h of treatment.

### 2.5. Differentially Expressed Genes

Changes in gene expression caused by the 12 h-treatment with juniper berry extract were examined by the cDNA representational difference analysis (RDA) to detect overexpressed genes in treated cells in comparison to non-treated control cells. This polymerase chain reaction-based, qualitative technique is able to detect very small mRNA-level differences in gene expression. The gene expression was analyzed at the time point when the amount of p53 starts to accumulate but there are no detectable apoptotic features of cell death.

**Figure 4:**
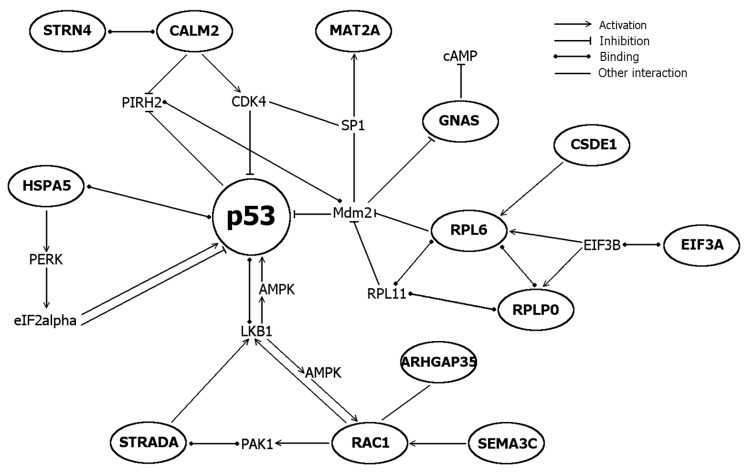
Interactions of proteins encoded by differentially expressed genes and p53 (in circles). Interactions were assessed by STRING 9.1 database accompanied with literature search. More detailed list of functions and references for proteins, see Table 4.

**Figure 5:**
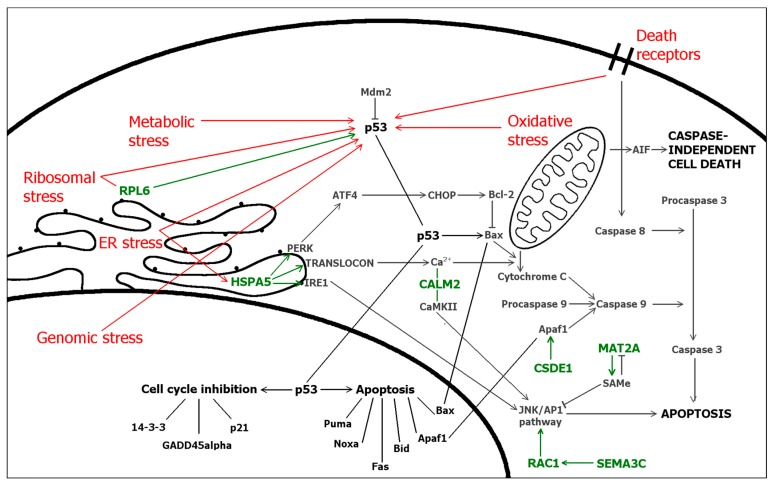
Cellular stress responses via p53 (**red**) and cell death pathways in the cell. In addition, interactions and functions of proteins encoded by differentially expressed genes (**green**) in related pathways. For more detailed functions of proteins, see Table 4. Bold lines represent plasma and nuclear membranes.

Twenty one overexpressed protein-coding genes were identified by BLAST search (Table 2). To clarify the varied nomenclature of genes and proteins, their preferred names and synonyms are listed in Table 3. To analyze the functions of proteins encoded by differentially expressed genes, they were assessed using literature search and UniProt KB and SwissProt databases (Table 4). Proteins were subdivided to seven different functional groups: cell death and cell survival proteins, cell cycle proteins, cellular stress proteins, cell shape, motility and polarity proteins, protein synthesis proteins, Ca^2+^-signaling proteins and proteins with enzymatic and protein-protein interactions. Further, the interactions of proteins with each other, p53 and cell death or survival were refined by STRING analysis accompanied with literature search (Figure 4 and Figure 5).

At the time when these genes were detected, the cells contained both pro-apoptotic and pro-survival signals and several proteins operating bifunctionally in cell survival and death either activating or inhibiting, i.e., CALM2 (calmodulin) and RAC1 (Rac1/TC25). Interestingly, genes related to ER stress (*CALM2*, *HSPA5*, *YKT6*), ribosomal stress (RPL6, RPLP0) and genomic stress (MORF4L1) were over-expressed. Direct interactions with p53 were found for ER stress sensor HSPA5 (BiP, GRP78), which is suggested to inactivate p53 via binding [23] and for ribosomal protein RPL6, which activates 53 via inhibiting Mdm2 [43]. STRADA and RAC1 modulate activations of p53 via LKB1. STRADA mediates G1 cell cycle arrest [44] whereas RAC1 promotes cell survival via PAKs [54]. The major regulator of calcium-mediated signaling CALM2 (calmodulin) modulates cell survival and death via different pathways, including JNK-pathway [28]. Under cellular stress, CSDE1 (UNR) helps eIFs (e.g., eIF3) to initiate internal ribosomal entry site (IRES)-mediated translation and one of its translational targets is a proapoptotic protein Apaf1 [29].

Interactions of seven identified genes with cellular stress, p53 and apoptotic cell death are elucidated in Figure 5. Results showed the expression of several cellular stress-induced genes responding to DNA damage (MORF4L1), ER stress (CALM2, HSPA5, YKT6) and ribosomal stress (RPL6, RPLP0). Together with genes modulating cell survival (e.g., MAT2A, SEMA3C), cell cycle and (e.g., ARHGAP35, STRADA, STRN4) and cell death (CSDE1, MORF4L1, RPLP0) these results suggest the cellular stress, especially ER stress, response mediated by Ca^2+^ (CALM2, HSPA5, STRN4).

## 3. Discussion

Numerous plant phenolics have been discovered to cause p53 accumulation in cells and this effect has been detected also in studies with flavonoid-rich plant extracts. Plant extracts are complicated mixtures of numerous bioactive compounds, which makes their testing challenging. In the present study, we have shown that the juniper extract can induce the p53 translocation from the cytosol into the nucleus and this occurred before the DNA fragmentation and cell death in the human neuroblastoma SH-SY5Y cells. The cleavage of DNA molecule into smaller fragments was detected 24, 48 and 72 h after the treatment while the most abundant accumulation and nuclear translocation of p53 occurred already before the DNA damage after 24–48 h. The breakage of DNA is one of the final steps of apoptotic cell death [26], and it is described as a hallmark of apoptosis [27]. Therefore, the p53-mediated DNA fragmentation suggests that the juniper berry extract activated cell death was mediated by active apoptotic mechanisms. Here, these changes were seen already at the early time point before a fully activated cell death when no caspase activity was detected but p53 started to be activated.

The human neuroblastoma cells are shown to possess sensitivity on toxicity treatments [25] and therefore chosen for testing of plant extracts in our studies [13]. The data concerning the role of p53 in neuroblastoma is controversial but the SH-SY5Y cell line contains wild-type p53 capable to transcription activity [58,59,60]. Mdm2 is the cytosolic main inactivator of p53 and the translocation of p53 in nucleus requires a breakage of a Mdm2-p53 complex accompanied with acetylation and phosphorylation [61]. Pro-apoptotic target genes of p53 include Pum, Noxa, Bax and Bid [62] but the major transcription target is p21 which contribute cell cycle arrest in G1 and G2 phases [63]. Transcription-independent actions of p53 include the interactions of p53 with different proteins in cytosol. For example p53 interacts with anti-apoptotic proteins Bcl-2 and Bcl-xL to enable pro-apoptotic proteins Bax and Bid to translocate on mitochondrial outer membrane [64].

To understand the molecular mechanisms related to the observed cell death, differentially expressed genes were identified and their functions analyzed. One aim of differential expression analysis was to discover the specific genes involved in the cellular response for the juniper extract. After treatment of neuroblastoma cells with 10 μg/mL of juniper berry extract, differentially expressed genes were analyzed by cDNA RDA method. Gene expression was evaluated 12 h after treatment with juniper berry extract, which reveals the upregulated genes before p53-mediated and DNA fragmentation-related cell death occurred. We detected twenty-one genes upregulated in response to the juniper extract (Table 2). The genes were classified to different functional groups (Table 4) to alleviate their analysis. The functions of detected genes were involved in cell death and survival (e.g., apoptosis and autophagy), cellular stress, cell cycle regulation, Ca^2+^-signaling, protein synthesis and protein-protein interactions.

Endoplasmic reticulum (ER) stress is the cellular response to the toxic or harmful stimulus. With this response the cells try to survive dangerous period and after removing the toxic stimulus, the cells restore normal activity. However, when the stimulus is longer, the cells die via apoptosis. Cells exploit ER stress caused by i.e., oxidative stress [65] to reduce misfolded and aggregated proteins, which is reported to trigger cyclosporine A-induced autophagy and apoptosis in malignant glioma cells with the inhibition mTOR/p70S6K1 pathway [66]. ER stress-induced glucose responsive protein BiP/GRP78 (HSPA5) is a heat shock protein, which belongs to chaperones; proteins involved in folding of macromolecules and expressed by ER stress [30]. BiP inhibits unfolded protein response (UPR) pathway and mediates both prosurvival and proapoptotic effects, respectively. It binds overexpressed wild-type p53 in nasopharyngeal carcinoma cells [23] and inhibits apoptosis via PERK/eIF2/NF-κB pathway [32]. In addition, it activates caspase 12 [67] and PERK/eIF2/ATF4/CHOP-mediated apoptosis [32].

Among other functions, ER is involved in Ca^2+^-homeostasis. Three overexpressed genes (CALM2, HSPA5, STRN4) involved in Ca^2+^-signaling and homeostasis advocate the role of calcium in stress response induced by the juniper berry extract. CALM2 (calmodulin) is a major regulator of calcium-mediated signaling, including the regulation of CaMKII-mediated apoptosis via JNK-pathway [28]. Interestingly, Rac1 has been found to promote JNK-mediated apoptosis as well [36] and SEMA3C (Sema E) is one of its activators [68]. Another overexpressed gene *STRN4* (zinedin) regulates calmodulin-mediated signaling [57] and is claimed to act as a sensor for concentration changes of Ca^2+^ [69]. Based on literature analysis and functional annotation of the list of genes, the juniper extract induces ER stress accompanied with accumulation of p53 in neuroblastoma cells. We also found that the apoptosis-related genes were activated by the juniper berry extract. Therefore, based on the gene expression profiling it is suggested that the treatment with 10 μg/mL of juniper berry extract induces ER stress in the neuroblastoma cells.

Recent studies have revealed that some of the over-expressed proteins comprise specific cellular stress, cell survival or death-mediating mechanisms beyond their well-known main functions (Table 4). Ribosomal proteins encoded by RPLP0 and RPL6 are involved in protein translation but they also possess extra-ribosomal functions. The upregulated RPL6 enhances cell growth and cell cycle progression [70] and RPLP0 has been found to by up-regulated after induction with doxorubicin and TRAIL [37]. The upregulated STRADA encodes STE20-related kinase adapter protein alpha (STRAD), which activates the tumour suppressor LKB1—A kinase involved in cell polarity, energy metabolism and cell growth [71]. LKB1 regulates cellular responses via different effectors and pathways including AMP-activated protein kinase (AMPK) activation after metabolic stress [72] leading to activation mTOR pathway and p53/p21-mediated cell cycle arrest and cell survival/apoptosis. Other tumour suppressor function of LKB1 involves the inhibition of oncogenic protein Yap [73].

In order to understand the cell death-inducing actions of aqueous juniper berry extract, we analyzed also its phenolic composition. To the best of our knowledge, this is the first report on the phenolic composition of an aqueous juniper berry extract. Six flavones, five flavonols, one flavanol, two phenolic acids and one biflavonoid were identified. We excluded the presence of volatile oil components by gas chromatography-mass spectrometry (GC–MS) method, because among phenolics volatile components of plants have shown to affect cellular mechanisms. Therefore, it is assumed that the aqueous nonvolatile oil containing juniper extract would be safer due to its lower toxicity in comparison with the volatile oil containing preparations. Otherwise, similar profiles of identified compounds have been detected by Innocenti et al. [9] with ethanol extract and Miceli et al. [18] with methanol extract. All of the identified compounds have been reported to possess different biological activities. Therefore, it is likely that their effects might explain also our observations partly.

Since the effects of juniper berry extract shown in this study might be explained by the activities of single phenolics, and the particular treatment concentrations were calculated for each phenolic compound found in the composition analysis (Table 1). Phenolic concentrations of our extract varied from 2.5 to 38.1 nM for single compounds. Literature search emphasized on neuroblastoma cells showed that cell death, cell cycle, DNA fragmentation and p53-related activities were described for identified compounds but usually at very high 100–1000-fold larger concentrations than in our study. The present results indicate that there might be synergistic or combinatorial effects involved explaining our results. e.g., 50 µM of apigenin decreased the SH-SY5Y cell viability after 24 h-treatment and increased the amount of subG1 apoptotic SK-N-DZ cells, caspase 3, caspase 8 and the proapoptotic ratio of Bax:Bcl-2 [74]. Apigenin concentration 1 µM decreased cell viability of SH-SY5Y cells 20% after 24 h-treatment but the increase of the ratio Bax:Bcl-2 and caspases 3 and 9 were detected with 50 µM-treatment [75]. In addition, quercetin has shown to activate wild-type p53 by breaking the Mdm2-p53 interaction [76].

There are only a few reports in literature to describe the biological effects of juniper berry extract. Naturally occurring plant phenolics and extracts are known to possess both antioxidant and pro-oxidant properties. The juniper berry extract used in this study has been evaluated for its antioxidant and pro-oxidant activity in our own laboratory [14,15]. It was shown that the extract possessed the pro-oxidant activity to stimulate protein degradation, and the antioxidant activity to chelate iron and to scavenge hydroxyl radicals. These properties might be involved in the cell death-inducing effects shown to plant phenolics. Tunón et al. [7] have tested traditional Swedish medicinal plants for their anti-inflammatory properties based on the ethnopharmacological use of plants. They showed that the extract from juniper berries possessed moderate inhibiting activity in both prostaglandin and platelet activating factor (PAF)-induced exocytosis tests in vitro.

The present results suggest that the juniper extract activates ER stress pathway in the human SH-SY5Y neuroblastoma cells accompanied with the increased amount and translocation of p53 from the cytosol to the nucleus, the expression of specific cell cycle and cell survival regulating genes, the DNA fragmentation and the apoptotic cell death. This activation was seen already before the caspase activity could be clearly detected. Therefore, it is concluded that the juniper berry extract is able to reactivate the impaired p53 function and apoptosis typically found in the human neuroblastoma cells. To the best of our knowledge, this is the first report of the phenolic composition of aqueous juniper berry extract. When the traditional use of plant extracts—including juniper berries—is often based on the aqueous extracts, these results improve the understanding of the possible mechanisms behind the traditional use of them.

## 4. Materials and Methods

### 4.1. Plant Material, Solvents and Reference Substances

Ripe and dried berries of juniper (*Juniperus communis* L.) cultivated for spice were commercially obtained from Paulig Ltd., Helsinki, Finland [77]. Details of origin or cultivation information are not available for the test material. Acetonitrile and formic acid were of high performance liquid chromatography (HPLC) or LC-MS grade. Flavonoids and phenolic acids such as amentoflavone, apigenin-7-*O*-glucoside, hyperoside, kaempferol-3-*O*-glucoside, rutin, protocatechuic acid and rosmarinic acid were purchased from Extrasynthese (Genay Cedex, France).

### 4.2. Aqueous Extraction of Juniper Berries

Ground juniper berries were extracted as described previously [15]. In brief, the extraction was performed by boiling water during three-hour hydrodistillation, resulting in removal of volatile oil. Hydrodistillation was performed using a Clevenger-type apparatus reported in European Pharmacopoeia [78]. The aqueous extract was filtrated, freeze-dried and stored at 4 °C. The yield was 422 mg/g of ground berries [15].

### 4.3. Analysis of Volatile Oils by Gas Chromatography-Mass Spectrometry (GC–MS)

Volatile oils were removed from the extract during the extraction process by hydrodistillation. The absence of volatile oil compounds was verified from hexane and ethyl acetate extracts of aqueous juniper extract (50 mg/mL) by gas chromatography-mass spectrometry (GC–MS). Analyses were carried out with GC–MS on a semipolar NB-54 column (HNU-Nordion Ltd., Helsinki, Finland). Briefly, the column was combined to HP5890 GC coupled to an HP5970 quadrupole mass selective detector, and helium was used as a carrier gas. Column temperature shifted during analyses from 50 to 250 °C at 10 °C/min.

### 4.4. Analysis of Phenolic Composition by High Performance Liquid Chromatography (HPLC)

The analyses were carried out using a Waters HPLC system (Waters Corp., Millford, MA, USA), consisting of a Millenium chromatography manager, 717 autosampler, 2996 photodiode array (PDA) detector, and 600 controller and a pump connected to an in-line degasser at room temperature. Compounds of juniper berry extract were separated in Phenomenex Synergi Fusion-RP column, 150 mm × 4.6 mm, 4 µm particles (Phenomenex, Torrance, CA, USA), using 0.1% formic acid (A) and acetonitrile (B) as solvents. A gradient elution with a flow rate of 0.4 mL/min was 0–5 min, 5% B; 10 min, 90% A and 10% B; 15–18 min, 75% A and 25% B; 25–28 min, 65% A and 35% B; and 35–40 min, 100% B. The phenolic composition was determined by comparing the retention data and absorption maxima of the UV spectra (200–500 nm) of reference compounds with the values of sample components and earlier published data in literature.

### 4.5. LC-MS-UV Systems and Analyses

High performance liquid chromatography (HPLC) analyses were performed on Agilent 1100 HPLC system (Agilent Technologies, Santa Clara, CA, USA) fitted with the same column as in the phenolic composition analysis, and by using corresponding mobile phases, gradient elution and flow-rate, with an injection volume of 40 μL at room temperature. The UV detection was made at 280 and 350 nm. An Agilent 6410 triple quadrupole MS with electrospray ion (ESI) source was used in negative ion mode.

In the MS identification, full scan ESI-MS^−^ spectra were acquired at a mass range of *m*/*z* 100–800 and at fragmentor voltage of 135 V. Juniper extract components were identified by comparing HPLC retention times, UV-vis spectra and MS fragmentation patterns with those of pure substances, and the data published in the literature.

In multiple reaction monitoring (MRM) technique, the fragmentation energy for parent → product ion (*m*/*z*) transitions were used at a range between 120 and 210 V for pure phenolic acids and flavonoids. Quantitation of sample components was based on five-point calibration curves (*r*^2^ = 0.991–0.999) prepared for reference substances. Other phenolics, not available as pure compounds, were quantified by using rutin as a standard.

Separation in ultra-performance liquid chromatography (UPLC) analyses was tested under different conditions regarding column, solvent A, gradient elution and flow rate. The analyses were performed on Waters Acquity UPLC™ combined with Waters QTOF Premier MS by using an Acquity™ BEH C18 column (100 mm × 2.1 mm, 1.7 µm). Gradient solvent system consisted of water/acetonitrile/formic acid (95:5:0.1, solvent A) and acetonitrile (solvent B). The proportion of B increased from 15% to 50% in 17.5 min and to 100% in 2.5 min. The flow rate was 0.3 mL/min and injection volume 5 µL. MS data were collected in ESI^−^ mode at a mass range of *m*/*z* 100–1000, and leucine enkephaline was used as the lock spray reagent.

### 4.6. Cell Culture and Treatments

Human SH-SY5Y neuroblastoma cells were cultured in Dulbeccos’s modified eagle medium: nutrient mixture F-12 (1:1) containing 15 mM HEPES buffer and l-glutamine and supplemented with 15% heat-inactivated foetal bovine serum, antibiotic mixture of penicillin (170 U/mL) and streptomycin (170 µg/mL) and 1% non-essential amino acids. Continuous culturing of cells was performed at 37 °C in a humidified atmosphere containing 5% CO_2_ in air. Cells were treated on 6 cm plates for DNA fragmentation assay and Western blot analysis, and on 10 cm plates for cDNA-RDA assay. For cell experiments, the juniper berry extract was dissolved in mQ-H_2_O at the concentration of 50 mg/mL.

### 4.7. Preparation of Protein Samples for Western Blot Analysis

The SH-SY5Y cells were collected using a cell scraper at different time points after treatment with 10 μg/mL of juniper berry extract, and spun down by centrifugation at 5000× *g* for 5 min. Cell pellets were stored at −80 °C until nuclear and cytoplasmic proteins were extracted by using a commercial kit (ProteoJET Cytoplasmic and Nuclear Protein Extraction Kit, Fermentas, Espoo, Finland) according to manufacturer’s instructions. Briefly, cell lysis and cytoplasmic protein extraction were performed by incubating cells with a cell lysis buffer supplemented with protease inhibitor. Cytoplasmic proteins were separated from nuclei by centrifugation at 1000× *g* for 10 min. Nuclear proteins were extracted from washed nuclei with nuclei lysis reagent by shaking samples at 1200 rpm for 15 min and separating proteins by centrifugation at 16,000× *g* for 15 min. All collection and extraction steps were performed on ice or at 4 °C. Protein samples were stored at −80 °C until protein amounts were determined by a colorimetric bicinchoninic acid (BCA) assay (Thermo Scientific, Waltham, MA, USA).

### 4.8. Western Blot Analysis of Cytoplasmic and Nuclear p53

Western blot analysis was performed as previously described by Lantto et al. [13]. Cytoplasmic proteins (15 μg) and nuclear proteins (30 μg) were first separated in 12% SDS-PAGE gel and transferred onto nitrocellulose membrane. Nonspecific binding of antibodies was blocked by 5% non-fat milk powder before exposing the membrane with a monoclonal p53 and β-actin primary antibodies (DO-7, Novocastra, Hämeenlinna, Finland; A1973, Sigma, Helsinki, Finland). To enable the visualization of proteins, the membranes were exposed to a horseradish peroxidase (HRP)-conjugated secondary antibody (HAF007, R&D Systems, Abingdon, UK) and a chemiluminescence reaction was induced by HRP-substrate (SuperSignal West Pico, Thermo Scientific, IL, USA). Proteins were detected and analyzed with GeneGnome system and GeneTools programme (Syngene, Frederick, MD, USA).

### 4.9. DNA Fragmentation

DNA fragmentation was determined by agarose gel electrophoresis as previously described [24,79], with minor modifications. In brief, SH-SY5Y cells were treated with 10 µg/mL of juniper berry extract. Both floating and attached cells were collected at different time points and centrifuged for 5 min at 5000× *g* at 37 °C. The pellets were resuspended in lysis buffer (0.2% Triton X-100; 10 mM Tris-HCl, pH 7.5, and 10 mM EDTA in water) for 20 min on ice. After a centrifugation of 20 min at 16,000× *g* at 4 °C, the supernatants were collected and incubated with RNAse enzyme (final concentration of 100 µg/mL) for 1 h at 37 °C. DNA was purified with phenol/chloroform and precipitated with 100% ethanol overnight at −20 °C. Precipitated DNA was collected by centrifugation for 30 min at 12,000× *g* at room temperature (RT) and washed with 70% ethanol. The pellets were dissolved in TE buffer (10 mM Tris-HCl, pH 8.0, and 10 mM EDTA in water) and mixed with DNA loading dye for electrophoresis. DNA fragments were separated in 2% agarose gel in TAE buffer (40 mM Tris acetate, 2 mM EDTA, pH 8,5) for 2 h 40 min using a voltage of 100 V. The image was photographed with a charge coupled device (CCD) camera (UltraLum, Claremont, CA, USA) under UV light.

### 4.10. cDNA Representational Difference Analysis (RDA)

Total RNA was isolated from juniper berry extract-treated and untreated cell samples by TRIzol-method (Gibco BRL, Gaithersburg, MD, USA). The total RNA samples were stored at −80 °C until the concentration, purity and integrity of total RNA were determined by 1.5% agarose gel and spectrophotometry. Double-stranded cDNA was synthesised from the total RNA by SuperScript™ Double-Stranded cDNA Synthesis kit (Invitrogen, Gaithersburg, MD, USA) according to manufacturer’s instructions. Representational difference analysis was performed as previously described by Hubank et al. [80] with some modifications. The ds-cDNA was digested with DpnII-enzyme (R0543S, BioLabs, Ipswich, MA, USA) and desalted R-Bgl-12/24 linkers (TAG Copenhagen, Frederiksberg, Denmark) were ligated to enable the PCR amplification with R-Bgl-24 primer. PCR products were cut by DpnII-enzyme to remove R-linkers and to produce driver sample which represents gene expression of untreated control cells. Tester sample representing gene expression of juniper berry extract-treated cells was prepared from cut PCR products by purifying R-linkers from the samples and ligating J-Bgl-12/24 linkers (TAG Copenhagen). The first difference products (DP1) were generated by hybridizing a mixture of tester and driver in a ratio of 1:100 followed by an exponential PCR amplification of tester:tester hybrids. Driver:tester and driver:driver hybrids were eliminated. To remove false annealing products, the second difference products (DP2) were produced. The DP1 were cut, ligated to N-Bgl-12/24 linkers (TAG Copenhagen) and hybridized with the driver at the ratio of 1:2000. After amplification by PCR the DP2 were cut with DpnII-enzyme, separated and purified by gel electrophoresis and ligated with BamHI-digested pGEM vector (Fermentas). The DP2 were cloned by transferring vectors into *E. coli* DH10B (TOP10) (Invitrogen) by electroporation. Plasmids were purified and the plasmid DNA analyzed by cycle sequencing (PE Applied Biosystems, 3730 DNA Analyzer, Tartu, Estonia) using M13 forward primers (BigDye, Applied Biosystems, Carlsbad, CA, USA).

### 4.11. Statistical Analysis

The western blot results are shown as arithmetic means ± SEM from three independent experiments. Comparisons between untreated control cells and treated cells were evaluated using a one-tailed Student’s *t*-test, *p* < 0.05 was defined as significant. Morphological changes of cells are shown as an arithmetic mean ± SD complemented with a median from three independent experiments.

## Figures and Tables

**Table 1:** Identification and quantification of phenolic compounds in aqueous juniper berry extract.

Peak	Compounds	Rt min	^a^ λ_max_ nm	*M*w	^b^ ESI-MS^−^ Ions *m*/*z*	MRM Ions	Amounts in Extract µg/g	Amounts in Treatments (10 µg/mL of Extract) nM (ng/mL)	References
1	Protocatechuic acid	11.11	260, 293	154	153, 109	153→109	412 ^d^	26.75 (4.12)	[16]
2	Catechin	13.69	280	290	289, 245, 205	-	406 ^e^	14.00 (4.06)	[17]
3	Gossypetin-hexoside-pentoside	15.07	271, 367	612	611, 479	-	155 ^e^	2.53 (1.55)	[18]
4	Rutin	20.97	255, 354	610	609	609→301	1333 ^d^	21.85 (13.33)	[9]
5	Hyperoside	21.14	255, 353	464	463, 301	463→300	561 ^d^	12.09 (5.61)	[9]
6	Quercetin pentoside	24.09	275, 341	434	433, 301	-	174 ^e^	4.01 (1.74)	[18]
7	Isoscutellarein-8-*O*-hexoside	24.72	276, 303, 327	448	447, 285, 895 ^c^	-	427 ^e^	9.53 (4.27)	[9,18,19]
8	Hypolaetin-7-*O*-pentoside	25.00	275, 342	434	433, 301, 867 ^c^	-	887 ^e^	20.44 (8.87)	[9,18,19]
9	Apigenin-7-*O*-glucoside	25.63	267, 338	432	431, 269	431→268	1646 ^d^	38.10 (16.46)	[9,18]
10	Luteolin pentoside	26.16	273, 347	418	417, 285	-	109 ^e^	2.61 (1.09)	[9]
11	Hypolaetin hexoside	26.79	257, 343	464	463, 301, 867 ^c^	-	305 ^e^	6.57 (3.15)	[9]
12	Isoscutellarein-7-*O*-pentoside	27.25	275, 304, 326	418	417, 285, 835 ^c^	-	798 ^e^	19.09 (7.98)	[9,18]
13	Kaempferol-3-*O*-glucoside	27.38	266, 346	448	447, 285	447→284	250 ^d^	5.58 (2.50)	[17]
14	Rosmarinic acid	27.56	329	360	359, 161	359→161	103 ^d^	2.86 (1.03)	[20]]
15	Amentoflavone	37.40	268, 340	538	537	537→375	167 ^d^	3.10 (1.67)	[9,18]

^a^ High performance liquid chromatography (HPLC)-DAD; ^b^ [M − 1] (100%), full scan mode; ^c^ detected by ESI-QTOF-MS^−^; ^d^ quantitation based on calibration curves; ^e^ calculated as rutin. References [9,21,22,23,24,25] et al. 2006. ESI: Electrospray ion; QTOF: Quadrupole time of flight; MS: Mass spectrometry; MRM: Multiple reaction monitoring; Rt: running time; *M*w: molecular weight.

**Table 2:** Differentially expressed genes and predicted proteins encoded by them. Differentially expressed genes were detected and sequenced from the purified mRNA of SH-SY5Y cells after 12 h treatment with 10 μg/mL of juniper berry extract by cDNA-RDA (representational difference analysis) method. Sequences were analyzed in BLASTN 2.2.27+ using human G + T databases. NM: accession number.

Gene	Accession Number	Score	*E*-Value	Length
*ALKBH5*	NM_017758	706	0.0	988
*ARHGAP35*	NM_004491	771	0.0	948
*CALM2*	NM_001743	499	2 × 10^−138^	663
*CSDE1*	NM_001242893+	627	1 × 10^−176^	720
*EIF3A*	NM_003750	508	4 × 10^−141^	706
*GNAS*	NM_003259+	669	0.0	899
*HSPA5*	NM_005347	682	0.0	683
*ITFG3*	NM_032039	647	0.0	939
*MAT2A*	NM_005911	363	4 × 10^−97^	363
*MORF4L1*	NM_006791	392	3 × 10^−106^	515
*PPAPDC1B*	NM_001102559+	453	2 × 10^−124^	833
*PRSS12*	NM_003619	508	5 × 10^−141^	859
*RAC1*	NM_006908+	536	2 × 10^−149^	908
*RPL6*	NM_001024662	508	5 × 10^−141^	869
*RPLP0*	NM_001002+	623	2 × 10^−175^	874
*SEMA3C*	NM_006379	616	3 × 10^−173^	907
*SF3B2*	NM_006842	544	1 × 10^−151^	932
*STRADA*	NM_153335	468	9 × 10^−129^	998
*STRN4*	NM_013403	608	3 × 10^−171^	663
*TTC37*	NM_014639	573	1 × 10^−160^	702
*YKT6*	NM_006555	621	5 × 10^−175^	792

**Table 3:** Nomenclature of differentially expressed genes and predicted proteins encoded by them. Gene synonyms are from the database of Hugo Gene Nomenclature Committee (HGNC) maintained by the European Bioinformatic Institute. Names of predicted proteins and their synonyms are from UniProtKB and Swiss-Prot databases.

Gene	Gene Full Name/Synonyms	Protein(s) Encoded/Synonyms
*ALKBH5*	Alkylating repair homolog 5 (*E. coli*)/ABH5	RNA demethylase ALKBH5
*ARHGAP35*	Rho GTPase activating protein 35/GRF1	Rho GTPase activating protein 35/GRF1/p190-A
*CALM2*	Calmodulin 2 (phosphorylase kinase, delta)/CAM2	Calmodulin/CaM
*CSDE1*	Cold shock domain containing E1/UNR	Cold shock domain-containing protein E1/UNR
*EIF3A*	Eukaryotic translation initiation factor 3, subunit A	Eukaryotic translation initiation factor 3, subunit A/eIF3a
*GNAS*	GNAS complex locus/NESP	ALEX
		Guanine nucleotide-binding protein G(s), subunit alpha isoform Xlas/Xlalpha
		Guanine nucleotide-binding protein G(s), subunit alpha isoform Short
		Neuroendocrine secretory protein 55/NESP55
*HSPA5*	Heat shock 70 kDa protein 5/GRP78	78 kDa glucose-regulated protein/GRP78/BiP
*ITFG3*	Integrin alpha FG-GAP repeat containing 3	ITFG3
*MAT2A*	Methionine adenosyltransferase II/MATA2	*S*-adenosylmethionine synthase, isoform type-2/MAT2
*MORF4L1*	Mortality factor 4 like 1/Eaf3/MRG15	Mortality factor 4-like protein 1/MRG15
*PPAPDC1B*	Phosphatidic acid phosphatase type 2, domain containing 1B/HTPAP	Phosphatide phosphatase PPAPDC1B/HTPAP/DPPL1
*PRSS12*	Protease, serine, 12 (neurotrypsin, motopsin)/MRT1	Neurotrypsin/Motopsin
*RAC1*	Ras-related C3 botulinum toxin substrate 1/p21-Rac1	p21-Rac1/TC25
*RPL6*	RPL6/TXREB1	60S ribosomal protein L6/TaxREB107
*RPLP0*	Ribosomal protein large P0	60S acidic ribosomal protein P0
*SEMA3C*	Sema domain, immunoglobulin domain (Ig)/SemE	Semaphorin-3C/Semaphorin E/Sema E
*SF3B2*	Splicing factor 3b, subunit 2, 145 kDa/Cus1	Splicing factor 3B subunit 2/SAP145
*STRADA*	STE20-related kinase adaptor alpha/LYK5 /STRAD	STRAD alpha
*STRN4*	Striatin, calmodulin-binding protein 4/ZIN	Sriatin-4/Zinedin
*TTC37*	Tetratricopeptide repeat domain 37/KIAA0372	TPR repeat protein 37/Ski3/Thespin
*YKT6*	YKT6 v-SNARE homolog (*S. cerevisiae*)/“R-SNARE”	Synaptobrevin homolog YKT6

**Table 4:** Functions of predicted proteins encoded by differentially expressed genes.

Functions	Genes	Specific Functions for Proteins Encoded by Differentially Expressed Genes
Cell death and cell survival	*CALM2*	Positive or negative regulator of apoptosis [1], regulator of autophagy [28]
	*CSDE1*	Activates IRES-mediated translation of proapoptotic protein Apaf1 during apoptosis [29]
	*HSPA5*	Activator of UPR-induced autophagy mediated by PERK/eIF2α/ATF4 pathway [30]
		Inhibits p53-dependent apoptosis via PERK/eIF2 activation [31] and apoptosis via PERK/eIF2/NF-κB [32]
		Enhances apoptosis via PERK/eIF2/ATF4/CHOP pathway [32]
	*MAT2A*	Downregulated in renal cancer cells/tissues increasing a cell survival via HO-1 [33]
	*MORF4L1*	Activator of tumour suppressor-mediated apoptosis^UP^ and nuclear ligand for proapoptotic agent [34]
	*RAC1*	Mediates cell survival via NF-κB when activated by SemaC342 and via PAKs25 and PI3K/Akt and p38/MAPK pathways [35]
		Promotes drug-induced apopotosis via JNK pathway [36]
	*RPLP0*	Upregulated during drug-induced apoptosis [37]
	*SEMA3C*	Mediates cell survival by activating Rac1 and NF-κB [32]
	*YKT6*	As a subunit of SNARE, mediates autophagy via comprising of phagophores [38]
Cell cycle	*ARHGAP35*	Positive regulator of cell cycle in lung carcinoma cells [39]
	*CALM2*	Controls cell cycle progression [28]
	*CSDE1*	Activates IRES-mediated translation of cell cycle regulator PITSLRE during mitosis [40]
	*MAT2A*	Inhibits the growth of liver cancer cells via SAMe [41]
	*MORF4L1*	Activator of tumour suppressor-mediated cell cycle arrest^UP^ accompanied with DNA repair [42]
	*RPL6*	Inhibition of cell cycle via stabilization of p53 by suppressing MDM2 activity [43]
	*STRADA*	Mediates G1 cell cycle arrest via activating tumor suppressor LKB1 [44]
	*STRN4*	Required for the completion of cytokinesis (abscission) while interacting with Mink1 [45]
	*YKT6*	Over-expression alters cell cycle by increasing mitotic index, DNA synthesis and decreasing cell size [46]
Cellular stress	*CALM2*	Regulates CaMKII, a significant mediator of ER stress induced apoptosis [28]
	*HSPA5*	Sensor and inducer of ER stress [30,47] and overexpressed itself during ER stress [47]
		Under ER stress, reduce translation and protein synthesis via PERK/eIF2 [5]
	*MORF4L1*	Responses to DNA damage repairing DNA double strand breaks [48,49]
	*RAC1*	Under cellular stress, mediates cell survival via PAKs [9], PI3K/Akt- and p38/MAPK-signaling [35]
	*RPL6*	Under ribosomal stress, stabilize p53 by suppressing MDM2 activity [43]
	*RPLP0*	Upregulated under cellular stress, e.g., ribosomal stress or drug-induced stress [12,50,51]
	*YKT6*	Under ER stress, mediates apoptosis as a subunit of SNARE [52]
Cell shape, motility and polarity	*ARHGAP35*	Inhibits cell motility and migration via folic acid receptor/cSrc/p190RhoGAP pathway [53]
	*RAC1*	Activator of cell motility via PAKs [54]
	*STRADA*	Regulation of cell polarity via activating LKB1 and via Rac1 and PAK1 [21]
Protein synthesis	*ALKBH5*	Involved in mRNA export and modifications [55]
	*CSDE1*	RNA binding protein^UP^ and IRES transacting factor (ITAF) [56]
		Under stress conditions, helps eIFs and other ITAFs to initiate alternative IRES-mediated protein synthesis [56]
	*EIF3A*	Protein synthesis (translation)^UP^
	*HSPA5*	Reduce translation and protein synthesis via PERK/eIF2 [32]
	*MORF4L1*	Regulates chromatin remodeling during transcription via HAT and HDAC complexes [43]
	*RPL6*	Ribosomal protein, protein synthesis (translation)^UP^
	*RPLP0*	Ribosomal protein, protein synthesis (translation)^UP^
	*SF3B2*	Splices mRNA^UP^
	*TTC37*	As a subunit of SKI complex, mediates RNA surveillance with PAF complex [57] and assists exosome in mRNA degradation^UP^
Ca^2+^-signaling	*CALM2*	Ca^2+^-binding protein, the major regulator of calcium-mediated signaling [28]
	*HSPA5*	Ca^2+^-dependent chaperone^UP^, involved in regulation of calcium leak during ER stress [47]
	*STRN4*	Regulates Ca^2+^-signaling via calmodulin [57], possibly a sensor responding to the concentration of calcium [4]
Enzymatic and protein-protein interactions	*CALM2*	Interacts calcium-dependently with hundreds of proteins [28], e.g., striatin family proteins including zinedin [4,22]
	*EIF3A*	Subunit of eukaryotic initiation factor eIF3 with eIF3b^UP^
	*HSPA5*	Binds and inactivate UPR-related signaling molecules PERK and ATF4 [30], interacts with p53 [23]
	*MAT2A*	Catalyzes the synthesis of biological methyl donor SAMe^UP^
	*MORF4L1*	Subunit of transcription regulators HAT and HDAC complexes mediating chromatin [24]
	*RPL0*	Ribosomal protein—A component of a ribosome large 60S subunit^HGNC^
	*RPLP0*	Ribosomal protein—A component of a ribosome large 60S subunit^HGNC^
	*STRADA*	Subunit of LKB1-STRADα-MO25 and STRADα-PAK1 complexes [21]
	*STRN4*	Regulatory subunit of PP2A in a STRIPAK complex [22], binds with several proteins including Mink1 [45] and calmodulin [4]
	*TTC37*	Subunit of a SKI complex which assists exosome in a mRNA degradation^UP^ and associate with PAF in transcription [57]
	*YKT6*	Subunit of SNARE complexes which function in tranportation^UP^

^HGNC^HUGO Gene Nomenclature Committee and ^UP^UniProt Database. Abbreviations: Akt = RAC-alpha serine/threonine-protein kinase; Apaf1 = apoptotic protease activating factor 1; ATF4 = activating transcription factor 4; CaMKII = Ca^2+^/calmodulin-dependent protein kinase; CHOP = C/EBP homology protein; cSrc = Proto-oncogene tyrosine-protein kinase Src; eIF = eukaryotic initiation factor; ER = endoplasmic reticulum; HAT = histone acetyltransferase; HDAC = histone deacetylase; HO-1 = heme oxygenase 1; IRES = internal ribosomal entry site; ITAF = IRES transacting factor; JNK = c-Jun *N*-terminal kinase; LKB1 = serine/threonine kinase 11; MAPK = mitogen-activated protein kinase; MDM2 = E3 ubiquitin-protein ligase; Mink1 = misshapen-like kinase 1; MO25 = armadillo repeat scaffolding-like protein; NF-κB = nuclear factor kappa B; PAF = RNA polymerase II associated factor; PAK = p21-activated kinase; PERK = pancreatic eIF2-alpha kinase; PI3K = phosphoinotiside 3-kinase; PITSLRE = p34Cdc2 -related protein kinase; PP2A = protein phosphatase 2A; SAMe = *S*-adenosyl-l-methionine; SKI = nuclear proto-oncoprotein; SNARE = soluble *N*-ethylmaleimide-sensitive-factor attachment protein receptor; STRIPAK = striatin-interacting phosphatase and kinase; UPR = unfolded protein response

## Abbreviations

CCD	charge coupled device
cDNA-RDA	cDNA representational difference analysis
QTOF	quadrupole time of flight
